# Magnetic Particle-Based Hybrid Platforms for Bioanalytical Sensors

**DOI:** 10.3390/s90402976

**Published:** 2009-04-23

**Authors:** Lia Stanciu, Yu-Ho Won, Mallikarjunarao Ganesana, Silvana Andreescu

**Affiliations:** 1 School of Materials Engineering, Purdue University, West Lafayette, IN 47907-2045, USA; E-Mail: ywon@purdue.edu (Y.-H.W.); 2 Department of Chemistry and Biomolecular Science, Clarkson University, Potsdam, NY 13699-5810, USA; E-Mail: ganesanm@clarkson.edu (M.G.)

**Keywords:** Biomagnetic materials, composite magnetic materials, bio-immobilization, biological sensors

## Abstract

Biomagnetic nano and microparticles platforms have attracted considerable interest in the field of biological sensors due to their interesting physico-chemical properties, high specific surface area, good mechanical stability and opportunities for generating magneto-switchable devices. This review discusses recent advances in the development and characterization of active biomagnetic nanoassemblies, their interaction with biological molecules and their use in bioanalytical sensors.

## 1. Introduction

In recent years, numerous types of magnetic particles of nanometer and micrometer dimensions and composites of these materials have become key components in different areas like catalysis, environmental remediation, the biomedical field and sensing devices, cell labeling and immunomagnetic separations, magnetic resonance imaging, targeted drug delivery, and bio-imaging [1–12]. Cell isolation, enzyme immobilization, drug targeting, waste water treatment are just few examples of such applications. These materials offer the potential of enhanced mechanical and catalytic properties when compared to their bulk counterparts. In the biosensors field, they have been used as bioimmobilization platforms, magnetic carriers of biomolecules, to separate or concentrate analytes and to control electrochemical processes at electrode surfaces [2,11,13–17]. The combination of magnetic manipulation with bioimmobilization, separation and detection capabilities have created unique opportunities for enhancing the performance of sensing devices.

Of the various magnetic materials reported in the literature for sensing purposes, iron oxides, mainly Fe_3_O_4_ have been the most widely used because of their simple preparation and superparamagnetic properties. Iron oxide nanoparticles (NPs) are also biocompatible, displaying no hemolytic activity or genotoxicity. The NPs can either be used for simple adsorption of biomolecules, or functionalized or encapsulated in polymers or silica materials to fabricate hybrid composites with increased biocompatibility and added functionalities. These materials, deposited onto the surface of a glassy carbon (GCE) electrode have provided performance characteristics comparable with those of Prussian Blue modified electrodes [14]. Fe_3_O_4_ particles have catalytic active sites for sensing hydrogen peroxide [14,18], which is the product of many enzymatic reactions (e.g. glucose oxidase, lactate dehydrogenase, cholesterol oxidase) and a key component in various chemical, biological, pharmaceutical, clinical, environmental, and food processes. Magnetic iron oxide NPs have gained a great deal of attention also due to their potential for providing control of electrochemical processes [19,20] and creating magneto-switchable devices [14,21]. Biological recognition elements can be attached to their surface to develop various catalytic and affinity sensors. The use of magnetic particles for this purpose brings a number of additional advantages such as control and transport of the bioassembly to a specific location onto/close to the transducer surface. Furthermore, they can be retained and removed with a magnet without affecting the transducer surface, thus creating possibilities for regeneration and reuse.

Extensive research papers and reviews covering the synthesis and characterization of various magnetic particles were published in literature in recent years. In this review, we focus on the most important and widely used magnetic particles-biomolecule hybrid systems for sensing applications, their use as electrode materials and immobilization matrices, and discuss the success and limitations of these materials in biological sensors.

## 2. Biomagnetic Particles Platforms

Significant progress has been made in the control of size, size distribution, shape, and chemical composition of magnetic particles in recent years [22–40]. These are readily available in different forms (Fe_2_O_3_, Fe_3_O_4_, Fe_3_S_4_, MO-Fe_2_O_3_ where M = Ni, Co, Zn etc) and can be purchased from several companies or prepared using established synthetic procedures [10]. However, bare magnetic particles tend to easily aggregate and therefore their use for bioanalytical purposes can be difficult. In addition, most of these particles are prepared in hexane or other organic solvents and therefore many biological applications involve particles with modified surfaces that render them biocompatible. For example, they can be surface functionalized with different organic or inorganic coatings in a core-shell format or be prepared in a composite form using various synthetic polymers and natural polysaccharides, like alginate cellulose and dextran [41–44], polyaniline [45–55], or silica glasses [45,56–68]. Tridimensional multilayer films of carbon nanotubes with Fe_2_O_3_ introduced in the nanotubes have also been reported for development of a magnetic electrochemical platform for enzyme sensors [69]. This material provided enhanced adsorption of protein molecules, controlled deposition of higher density of carbon nanotubes onto the electrode surface and improved electrochemical properties.

An elegant approach is to coat the particle surface with a gold shell [23,70–83], favouring protein binding by taking advantage of their gold affinity. Another interesting example is to coat the NPs with a porous, optically transparent sol-gel layer resulting in a multifunctional hybrid material in which the outer silica shell stabilizes the particles, induces biocompatibility and provides sites for surface modification with biological molecules, electronic mediators or fluorescent labels [84,85]. Such hybrid materials combining the properties of silica microspheres with the advantages of magnetic particles hold promise for additional applications. For example, multifunctional magnetic particles that incorporate chromophores can be easily manipulated through the use of an external magnetic field, while their position at a given time and place can be monitored using fluorescence methods [86,87]. Chromophores used for such applications included organic dyes such as rodhamine [84] as well as quantum dots, featuring narrow emission bandwidth, large two-photon absorption and continuous adsorption spectra [12,88–94].

**Figure 1.**
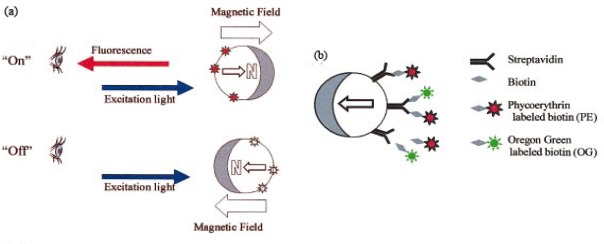
(a) An external magnetic field orients the aluminum-capped Mag-MOON, causing its fluorescent excitation and observed emission to blink on and off as it rotates; (b) Scheme of an assay for the measurement of relative concentration of biotin molecules. The biotin is labeled with two types of fluorescent dyes (reproduced with permission from reference [95])

Anker *et al.* [95] synthesized metal-capped magnetically-modulated nanoprobes (Mag-MOON), that have the capacity to rotate under changing magnetic fields and emit light fluxes in different orientations. In general, fluorescent NPs emit light uniformly in all directions. When coated with a metal layer the symmetry is disturbed and the particles start emitting different amounts of light in different orientations. The orientation of the particle can also be tracked in time. The use of Mag-MOON particles allows *in-situ* fast background subtraction and provides a significant increase of signal-to-noise ratio. Therefore, immunoassays with a variety of fluorescent labels can be designed. For example, fluorescent dyes have limitations in terms of signal-to-noise ratio when cellular responses are investigated. Mag-MOONs have the potential to increase the sensitivity of these assays when used in nanosensors that are designed to perform rapid single cell measurements. Immunoassays based on Mag-MOONs can be designed for protein detection. Briefly, the Mag-MOONs can be functionalized with molecular recognition elements or antibodies that can bind specifically analytes of interest. In a subsequent step a fluorescent tag is added to increase Mag-MOONs brightness and allow antigen detection (Figure 1).

Silica-coated magnetites were obtained using various synthetic procedures [93,94,96–101]. The thickness of the outer silica shell can be tuned by changing the concentration of the silica precursors during synthesis [84]. Zhao *et al.* [102] fabricated magnetic nanospheres covered with a mesoporous silica shell starting from uniform magnetite NPs, with a mean diameter of 120 nm. These were subsequently covered by a mesoporous silica coating via sol-gel polymerization using tetraetoxysilane (TEOS) and n-octadecyltrimethoxysilane (C18TMS). In the last step, a reduction of the hematite core under an atmosphere of H_2_ and N_2_ was performed, to lead to the formation of magnetic core/mesoporous silica shells with a narrow size distribution and a mean diameter of approximately 270 nm.

Lee, *et al*. [97] recently reported a one-pot synthesis of uniformly sized, non-agglomerated magnetic silica core-shell particles by using a reverse micelles procedure and alkoxide precursors. The synthesis was performed at 90 °C, which allowed maintaining the micelle structure. Two model enzymes (lipase and α-chemotrypsin) were crosslinked with glutaraldehyde (GA) and the results were compared with those obtained by covalent attachment on the NPs surface, through amine functionalization. As an alternative, biological molecules can be entrapped within the silica glass to generate a biologically active material with enhanced stability while providing magnetic properties for easy manipulation and control under a magnetic field. Preparation of silica coated iron oxide NPs with an average diameter of 5 – 7 nm has been described in the literature [96]. The size of the magnetic nanocomposite after the deposition of the silica was ∼ 53 nm.

## 3. Magnetic Activation of Redox Processes in Bioanalytical Sensors

In bioelectronic devices, such as biosensors and biofuel cells, the electrical contact between enzymes and the electrodes is essential [103–114]. The main challenge in achieving good electrical contact stays in the lack of direct communication between the redox (Reduction-Oxidation reaction) center of the biomolecule and the electrode surface. Solutions such as attachment of redox-relay groups to the enzyme, the use of diffusional electron mediators, or the immobilization in a redox polymer matrix have poor efficiency. Problems include the inappropriate orientation of enzyme in respect to the electrode, and conformational modifications of the protein structure. The conjugation of NPs with enzymes in hybrid devices holds promise for the development of novel and improved biosensing platforms and ensures electrical ‘wiring’ between the enzyme and the electrode. Redox-active units are used in a series of electrocatalytical and bioelectrocatalytical processes. Magnetic particles have been functionalized with redox units and subsequently used in reactions relevant for such processes. Examples of redox-active units that have been used to functionalize magnetic nano or micro particles include microperoxidase-11, pyrroloquinoline quinone, 2,3-dichloro-1,4-naphthoquinone, *N*-ferrocenylmethyl)aminohexanoic acid, or *N*-methyl-*N*”-(dodecanoic acid)-4,4′-bipyridinium [115,116]. When redox units-functionalized magnetic particles are attracted to the electrode surface with the help of a magnet placed underneath the electrode, the electrical contact between the redox units on the particles and the electrode is activated and the electrochemical response of the sensor is switched “ON”. In turn, when the magnet is placed on top of the electrode, the NPs are removed from the electrode surface, and the electrochemical response is switched “OFF” [117].

Redox relay units such as bipyridinium or ferrocene are also part of this set-up, and can serve as mediators for electron transfer between redox enzyme and the electrode [115]. This approach has been used for enzymes such as glucose oxidase (GOx) and nitrate reductase [118]. Under a magnetic field, ferrocene oxidizes to ferrocenyl cations. In turn, the ferrocenyl cations have an oxidizing effect on the redox center of the GOx. Removing the magnetic particles from the electrode also removes the electrical contact between the ferrocene and the electrode, inhibiting glucose oxidation. In the case of nitrate oxidase [115], bipyridinium was used as a reducing agent in the reduction process of nitrate to nitrite. When the bipyridinium functionalized magnetic NPs interacted with the electrode under an electrical field (E = − 0.7V), the nitrate-nitrite transformation was favored only when a magnet was positioned in the system in such a way that the NPs were attracted to the electrode. In the same time, the removal of the magnetic particles led to the electrocatalyzed reactions being switched off. Lactate dehydrogenase (LDH) was also used in a similar approach that included pyrroloquinoline quinone as a relay mediator for the activation/deactivation of the NADH-NAD^+^ transformation [119–121]. Selective dual biosensors were also designed with LDH and GOx and magnetic particles functionalized with monolayers of PQQ-NAD^+^ and ferrocene [122]. These were tested in both oxidation of glucose and inhibition of lactate oxidation when no electrical field was applied. When a small electrical field was applied (potential range: −0.13 < E < 0.13 V), the oxidation of lactate was enabled, while the oxidation of glucose was inhibited, since the potential range used does not favor the oxidation of ferrocene units.

## 4. Biofunctionalization of Magnetic Nanoparticles

Immobilization of enzymes, antibodies, oligonucleotides, and other biologically active compounds onto magnetic NP platforms is a key element in using these structures for biosensing purposes. Fabricating biofunctionalized magnetic materials containing a high amount of the biological element with high activity and stability is essential for the design of robust sensors that take advantage of the magnetic capabilities. Various routes for the fabrication of biofunctionalized magnetic NPs include traditional methods such as covalent binding, adsoprtion, specific affinity interactions, and entrapment in porous surface layers. In the following sections we describe representative procedures utilized for this purpose and provide selected examples reported in the literature.

### 4.1. Biofunctionalized Magnetic Nanoparticles Via Covalent Binding

The primary functionalization of iron oxide NPs with organic functionalities is the first step in the covalent binding of biomolecules to their surfaces. The drawbacks of this method stay in the restrictions derived from the biomolecule conformation being imposed by their orientation on the support upon binding. Functionalization of magnetic NPs with carboxyl, amino or hydroxyl groups prior to the covalent binding is traditionally used [6,39,123–138]. Hong *et al.* [139] functionalized magnetic nanogels through a carbodiimide activation procedure. In this case, polyacrylamide (PAM) coated Fe_3_O_4_ NPs and Hoffman degradation was used to obtain amino-functionalized magnetic nanogels with a 25 nm diameter. Subsequently, α-chymotrypsin was covalently attached to the nanogel’s surface. The enzymatic activity was largely retained and the reaction temperature and usable pH range were wider after the covalent binding as compared to the free enzyme. In the case of the covalent binding, the enzymatic activity was not dramatically affected by varying the pH between 5.8 and 8.9, while for the free enzyme, the enzymatic activity decreased to less than 50% at pH values between 8 and 8.9. The temperature was varied between 35 and 85 °C and the optimum temperature for the both free and covalently bound enzyme was determined to be at 35 °C, while above this temperature, a decrease in activity was observed in both cases. However, after 60 °C, the free enzyme lost all its activity, while the covalently bound enzyme still retained 60% of its activity. The enzymatic activity was retained after storage at 4 °C for 36 days. The magnetic composite was reusable, with 96.8% of the biological activity being maintained after 12 usage cycles. On the other hand, the affinity between the enzyme and the substrate was lower than that of the free enzyme. An explanation related to sterric effects of diffusion barriers of the substrate to the enzymatic active sites was proposed. More recently [140], the same authors used a hydrophilic polymer with free carboxyl groups to covalently immobilize α-chymotrypsin to the surface of magnetic iron oxide NPs, followed by *in-situ* polymerization with 1-ethyl-3-(3-dimethylaminepropyl) carbodiimide (EDC) used as a coupling agent. The enzyme retained 80% of its activity at 65 °C, 90% at 25 °C and the full enzymatic activity at 4 °C. The same long term storage stability and reusability as in the case of the amino functionalized nanogels were observed. Other enzymes covalently immobilized on the surface of magnetic NPs include GOx [138] and peroxidase [141,142], as well as cholesterol oxidase, lipase [143], trypsin and chymotrypsin [131,136,137,144–150]. Kuroiwa *et al.* [151] immobilized chitosanase on amylose-coated Fe_3_O_4_ NPs. The amylose provided hydroxyl groups on the NPs surface, which were then coupled with the amino groups of chitosanase via covalent binding. Fe_3_O_4_-chitosan NPs, exposing free amino groups on their surface were used to bind alcohol dehydrogenase via GA coupling, with about 48% retention of enzymatic activity [151]. The authors demonstrated the recovery of the enzyme by magnetic separation.

### 4.2. Biofunctionalized Magnetic Nanoparticles via Surface Adsorption

Core-shell NPs functionalized with polymers were investigated as supports for biomolecule immobilization. Mahmood *et al.* [152] immobilized lipase on magnetic NPs coated with a oleic acid-Pluronic^®^ (L-64) block copolymer. In this work, the copolymer was used to improve stability (i.e. reduce agglomeration). Up to 90% enzymatic activity of lipase was retained for seven cycles. This extended usability was attributed to hydrophobic interactions between the preatreated NPs and the copolymer. Shamim *et al*. [153] synthesized core-shell iron oxide NPs coated with poly-(*N*-isopropylacrylamide) (PNIPAM) and used them to adsorb bovine serum albumin (BSA) on their surface. Thiodiglycolic and 4-vinylaniline were used as surfactants. The core-shell NPs were thermosensitive. Above 32 °C, the structure of the poly-(NIPAM) changes from hydrophilic to hydrophobic. Due to this effect, at temperatures above 32 °C, the magnetic NPs shrink, and are able to adsorb a larger amount of bovin serum albumin (BSA). Lowering the temperature below 32 °C resulted in desorption of the protein. It has been shown that the protein adsorption takes place a larger extent through hydrogen bonding, and less through hydrophobic interactions. Peng *et al*. [154] fabricated Fe_3_O_4_ magnetic NPs with 10 nm in diameter through a chemical precipitation method and further used them for physical adsorption of BSA and lysozyme (LSZ) with partial retention of enzymatic activity. Desorption of both BSA and LSZ was also investigated at the isoelectric points of the enzymes. Kausik *et al.* [155] used Fe_3_O_4_ NPs prepared via a co-precipitation method to design a glucose sensor. The NPs dispersed in chitosan formed a film onto an indium-tin oxide (ITO) glass. Dispersion in chitosan helped preventing aggregation, while the NPs facilitated communication with the electrode surface. This biosensor had a rapid response time (5 s), good linearity (10 – 400 mg dL^−1^), good reproducibility, and high affinity towards glucose. The sensitivity was of 9.3 μA/(mg·dL·cm^2^) and the sensor was stable for up to eight weeks at 4 °C.

### 4.3. Entrapment of biomoleules in magnetic composites

Biomolecules can be entrapped within the different polymeric or silica shells used to form hybrid magnetic composites. For example, spherical silica coated Fe_3_O_4_ NPs have allowed stable entrapment of horseradish peroxidase (HRP), simultaneously with the formation of the silica layer [96]. This method resulted in biomagnetic catalysts characterized by a long-term stability with temperature up to 85 °C and pH change, as compared to the free enzyme. However, the entrapped enzyme could lose activity due to conformational changes in the silica matrix and also suffers from possible diffusion limitations of the substrate through the silica pores. The same methodology allowed immobilization of an antibody for the development of an immunoassay, for the quantitative determination of gentamicin with a detection limit of 160 ng/mL. More complex systems with entrapped enzymes combining different types of materials were also reported. Such an example is the use of iron oxide NPs with crosslinked enzyme molecules, which were then encapsulated into large pores of mesoporous silica to form a “hierarchically ordered, mesocellular” structure [145]. These nanocomposites were magnetically separable, highly loaded with enzyme, stable under harsh shaking conditions, resistant to different treatment procedures, and reusable.

### 4.4. Site specific bioimmobilization onto magnetic nanoparticles

Site specific immobilization onto magnetic particles is an attractive strategy for attaching biomolecules because it provides a favorable orientation for biorecognition events while avoiding conformational changes, and offering magnetic control of the entire assembly. As opposed to other methods, this strategy involving attachment at a specific pre-determined position eliminate the diffusion barriers or chemical bond formation that could affect the biological activity and, therefore, a lower detection limit and a fast response time could be expected for sensors fabricated based on this method. Johnson *et al.* [156] developed a method for immobilization of affinity-tagged dehalogenase on iron/iron oxide core-shell NPs through a biomimetic approach. The authors used cloned dehalogenase (DhlA) fusion proteins, with an affinity for either silica or iron oxide surfaces [157,158]. Three different DhLA recombinant enzymes were expressed and immobilized on the NPs surface. The enzymes were able to specifically bind to either iron oxide or silica. The DhLA enzymes tagged with iron oxide or silica affinity peptides showed a higher rate of adsorption onto the magnetic NPs when compared to the His-tagged protein. In another example, pre-activated iron oxide beads carrying Ni- iminodiacetic acid (IDA) complexes were used to immobilize a genetically-modified acetylcholinesterase (AChE), having engineered an hexa(histidine) tag. The 6His-AChE-Ni-NTA-NPs system was deposited onto the surface of a screen-printed electrode surface with a small magnet placed underneath the electrode. This biosensor was used for the detection of two organophosphorous pesticides, via AChE inhibition [159]. The main advantage of this method for inhibition assays is the very high sensitivity with detection limits of 10^−11^ M for chlorpyriphos-oxon and the easy reusability of the same electrode. The system could be easily adapted for integration into an autonomously operated magnetoswitchable device for monitoring AChE inhibiting activities.

## 5. Applications of Biomagnetic Materials in Sensing Technology

### 5.1. Enzyme Sensors

Enzymatic sensors based on various magnetic platforms, mainly iron oxides were designed with the advantages of offering high enzyme loading and control of the localization of the sensitive material through the use of a magnet allowing for the enzymatic reactions to occur in the close proximity of the transducer surface [159–163].

**Figure 2.**
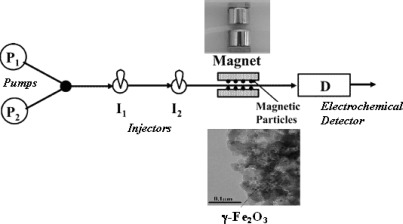
Schematic diagram of a magnetic microflow system based on GOx functionalized biomagnetic particles with electrochemical detection for the detection of glucose (adapted from reference [161] with permission from the American Chemical Society).

The transducing component is easily renewable and reusable. This is due to the ability to control the charging/discharging of its surface by application of a magnetic field, providing reusability of the same electrode for several analyses. Examples include sensors based on tyrosinase for the detection of phenol [162], yeast (YADH/NAD^+^) [164] for the detection of ethanol, GOx for glucose [163], and AChE for organophosphorus pesticides [151]. Enzyme-immobilized magnetic beads can be incorporated in a flow injection analysis (FIA) system as described by Kauffmann *et al.* [161]. Figure 2 shows the schematic diagram of a FIA assay for detection of glucose using glucose oxidase modified magnetic microparticles. Functionalized magnetic particles were injected into the FIA system and retained near the detector using a pair of two small permanent magnets. An amperometric system for the detection of glucose with glucose oxidase immobilized onto the magnetic particles was developed based on this principle. The porous biomagnetic particles were ferromagnetic spinnel type iron oxide (γ-Fe_2_O_3_) with the GOx immobilized after silanization with aminopropyltrietoxysilane followed by covalent binding using GA.

### 5.2. Immunosensors

**Figure 3.**
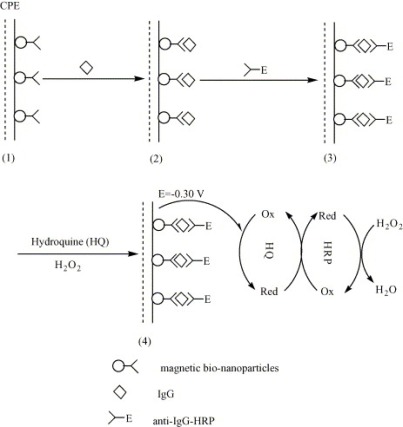
Preparation of the immunosensor and its application in IgG determination: (1) magnetic bio-nanoparticles containing anti-IgG were attached on the surface of carbon paste electrode in the presence of magnetic field; (2) incubation of the immunosensor with IgG solutions allowed formation of the anti-IgG/IgG complex on the electrode; (3) incubation of the immunosensor in HRP-labeled anti-IgG solutions allowed formation of anti-IgG/IgG/anti-IgG-HRP complex on the electrode; (4) hydroquinone and H_2_O_2_ were added and electrode-bound IgG was determined by amperometric measurements at an potential of - 300mV (vs. SCE) (reproduced with permission from reference [167]).

Magnetic particles functionalized with specific antibodies (Ab) have been used for the design of immunomagnetic sensors through the immobilization of the Ab-NPs assembly on the surface of an electrochemical transducer [3,165–167]. For these types of immunosensors, the immunomagnetic complex is magnetically attached to the surface of the screen printed electrode. The utilization of Ab-coated magnetic particles is efficient in overcoming the need of regeneration of the sensing surface and makes possible integration into automatic systems, difficult to achieve otherwise due to the obstacles in renewing the sensing surface. The immunocomplex is usually quantified through the use of enzyme labels, with the electrochemical detection of the enzyme reaction product after the complex is exposed to the enzymatic substrate, or through a fluorescent label followed by fluorescence detection. An example of amperometric immunosensing assay obtained by immobilizing the antibody onto a solid carbon paste electrode using core-shell magnetic NPs is shown in Figure 3 [167].

Different analytes were detected using this method, such as: rabbit IgG [168–170], non-pathogenic *E. coli* O157 [171], polychlorinated biphenyls (PCBs), the herbicide 2,4-D, atrazine [171–175] pesticides and bacterial pathogens [176,177]. For example, streptavidin activated magnetic microbeads were used for the immobilization of an antibody for atrazine, a small pesticide molecule, allowing for the determination of its concentration in biological samples, with a detection limit of 0.027 nmol L^−1^ [173]. In another work, immunomagnetic separation coupled with differential pulse voltammetry allowed detection of Arochlor 1248 PCB mixture with a detection limit of 0.4 ng/mL using screen-printed three electrode strips [178]. Other immunosensors with renewable electrode surface were reported for the detection of pathogens such as *Salmonella Typhimurium* [176,177]. Another interesting application of immunomagnetic NPs was for selectively concentrating traces of pathogenic bacteria (*Staphyloccocus saprophyticus* and *Staphyloccocus aureus*) via IgGs attached onto the particles surface [179]. It was found that these IgG-NPs conjugates can bind selectively to the cell membrane. Detection limits of as low as 3 × 10^5^ cfu/mL were achieved in aqueous solutions. Other specific examples of monodispersed bio-functional magnetic NPs for protein separation and pathogen detection were discussed recently by Gu *et al.* [180].

### 5.3. DNA sensors

DNA sensors with single-stranded (ss) oligodeoxynucleotides immobilized on electrode surfaces [181–183] via magnetic beads were also fabricated [184–190]. A three layer magnetic NPs structure with a gold surface, silica core and magnetic inner layer was functionalized with DNA and used for quantifying hybridization events [189]. It was found that upon hybridization with complementary oligonucleotides, this structure forms aggregates in the same way as Au NPs. In another work, surfactant-modified oligonucleotides were incorporated into the particle organic shell to create a DNA functionalized surface [188]. For example, monodisperse MnFe_2_O_4_ magnetic NPs were biofunctionalized with DNA using a combination of alkylphosphonate and ethoxylated fatty alcohols. This method is based on the affinity of alkylphosphonate for metal oxide surfaces. DNA amplification with magnetic primers in combination with electrochemical detection and enzyme labelling was used to develop a genomagnetic assay for detection of food pathogens such as *Salmonella* spp [190]. The electrode consisted of a graphite epoxy composite and streptavidin modified magnetic beads with immobilized DNA. Loaiza *et al.* [191] reported a sensitive method for isolation and detection of DNA from bacterial cells using disposable magnetic DNA sensors. Screen-printed gold electrodes with a 4 mm in diameter working electrode surface were used as amperometric transducers in this example.

**Figure 4.**
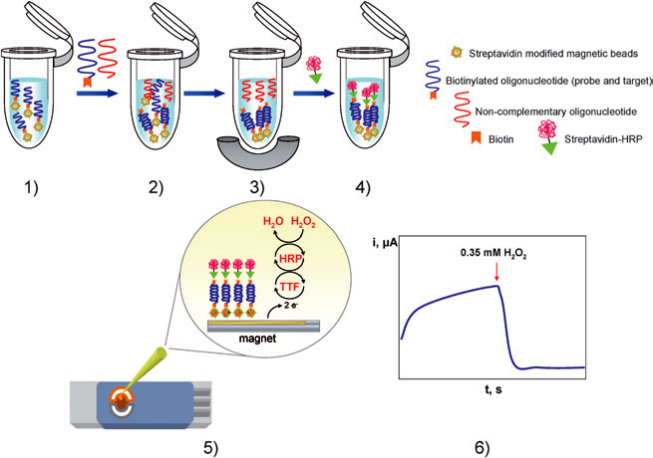
Schematic representation of the enzyme amplification protocol: (1) probe-modified magnetic beads washing step; (2) hybridization with the target lacZ gene probe; (3) hybrid-modified magnetic beads separation and non-complementary oligonucleotide extraction; (4) enzymatic labelling with streptavidin-HRP; (5) hybrid-modified magnetic beads deposition on the TTF-Au/SPEs; (6) amperometric detection of the mediated reduction of H_2_O_2_ with TTF (reproduced, with permission, from reference [191])

Figure 4 shows a schematic of the protocol used for enzyme amplification. The magnetic particles were functionalized with streptavidin. A 25-mer capture probe was subsequently attached, followed by the hybridization process. In the next step, streptavidin-peroxidase was attached and the functionalized magnetic particles were attached to the surface of the electrode through a magnet. The sensor was used for the detection of asymmetric DPCR amplified products obtained from *E. coli* bacterial cultures, with high speed, specificity and sensitivity. Another advantage of this method was elimination of false positive results, a drawback of conventional PCR analysis.

## 6. Conclusions

In this review, we have summarized the most recent developments in the field of biofunctionalized magnetic particles and their applications in bioanalytical sensors. Research in this field has focused mainly on the challenges of creating hybrid composite materials containing biomolecules, magnetic NPs and other inorganic components, and on the development of strategies that can be used to integrate these materials in functional sensing devices. Research in this direction involves scientific aspects of the preparation, structure and properties of magnetic systems and their functionalization with biological materials. This approach is unique in that it combines the high selectivity and specificity of biological processes with the high surface area and magnetic properties of magnetic particles. These properties have been used to provide control and orientation of the biomolecular recognition elements onto transducer surfaces as well as to enhance the response time, selectivity, sensitivity and stability of the sensor. These hybrid systems are the foundation of new generations of materials that could be used in the construction of biosensors, bioreactors, biofuel cells and in other biotechnological applications. The success of these research efforts is still dependent of the transferability of these materials into real life applications. The rapid developments in the field allowing successful fabrication and testing of various sensors designs based on biomagnetic assemblies illustrate the potential of this approach for further applications.

## References

[1] Luo X., Morrin A., Killard A.J., Smyth M.R. (2006). Application of nanoparticles in electrochemical sensors and biosensors. Electroanalysis.

[2] Khan R., Dhayal M. (2008). Electrochemical studies of novel chitosan/TiO_2_ bioactive electrode for biosensing application. Electrochem. Comm.

[3] Corti M., Lascialfari A., Micotti E., Castellano A., Donativi M., Quarta A., Cozzoli P.D., Manna L., Pellegrino T., Sangregorio C. (2008). Magnetic properties of novel superparamagnetic MRI contrast agents based on colloidal nanocrystals. J. Magn.Magn. Mater.

[4] Jun Y.W., Huh Y.M., Choi J.S., Song H.T., Kim S., Yoon S., Kim K.S., Shin J.S., Suh J.S., Cheon J. (2005). Nanoscale size effect of magnetic nanocrystals and their utilization for cancer diagnosis via magnetic resonance imaging. J. Am. Chem. Soc.

[5] Song H.T., Choi J.S., Huh Y.M., Kim S., Jun Y.W., Suh J.S., Cheon J. (2005). Surface Modulation of Magnetic Nanocrystals in the Development of highly efficient magnetic resonance probes for intracellular labeling. J. Am. Chem. Soc.

[6] Hanessian S., Grzyb J.A., Cengelli F., Juillerat-Jeanneret L. (2008). Synthesis of chemically functionalized superparamagnetic nanoparticles as delivery vectors for chemotherapeutic drugs. Bioorgan. Med. Chem.

[7] Liu T.Y., Hu S.H., Liu K.H., Liu D.M., Chen S.Y. (2008). Study on controlled drug permeation of magnetic-sensitive ferrogels: Effect of Fe3O4 and PVA. J. Control. Rel.

[8] Chen F.H., Gao Q., Ni J.Z. (2008). The grafting and release behavior of doxorubincin from Fe_3_O_4_@SiO_2_ core–shell structure nanoparticles via an acid cleaving amide bond: the potential for magnetic targeting drug delivery. Nanotechnology.

[9] Wu S.H., Lin Y.S., Hung Y., Chou Y.H., Hsu Y.H., Chang C., Mou C.Y. (2008). Multifunctional mesoporous silica nanoparticles for intracellular labeling and animal magnetic resonance imaging studies. Chembiochem.

[10] Safarik I., Safarikova M. (2002). Magnetic nanoparticles and biosciences. Monatsh. Chem.

[11] Li Y.F., Liu Z.M., Liu Y.Y., Yang Y.H., Shen G.L., Yu R.Q. (2006). A mediator-free phenol biosensor based on immobilizing tyrosinase to ZnO nanoparticles. Anal. Biochem.

[12] Insin N., Tracy J.B., Lee H., Zimmer J.P., Westervelt R.M., Bawendi M.G. (2008). Incorporation of iron oxide nanoparticles and quantum dots into silica microspheres. ACS Nano.

[13] Salimi A., Hallaj R., Soltanian S., Mamkhezri H. (2007). Nanomolar detection of hydrogen peroxide on glassy carbon electrode modified with electrodeposited cobalt oxide nanoparticles. Anal. Chim. Acta.

[14] Hrbac J., Halouzka V., Zboril R., Papadopoulos K., Triantis T. (2007). Carbon electrodes modified by nanoscopic iron(III) oxides to assemble chemical sensors for the hydrogen peroxide amperometric detection. Electroanalysis.

[15] Schachl K., Alemu H., Kalcher K., Jezkova J., Svancara I., Vytras K. (1997). Amperometric determination of hydrogen peroxide with a manganese dioxide-modified carbon paste electrode using flow injection analysis. Analyst.

[16] Yao S., Xu J., Wang Y., Chen X., Xu Y., Hu S. (2006). A highly sensitive hydrogen peroxide amperometric sensor based on MnO_2_ nanoparticles and dihexadecyl hydrogen phosphate composite film. Anal. Chim. Acta.

[17] Hermanek M., Zboril R., Medrik I., Pechousek J., Gregor C. (2007). Catalytic efficiency of iron(III) oxides in decomposition of hydrogen peroxide: competition between the surface area and crystallinity of nanoparticles. J. Am. Chem. Soc.

[18] Sljukic B., Banks C.E., Compton R.G. (2006). Iron oxide particles are the active sites for hydrogen peroxide sensing at multiwalled carbon nanotube modified electrodes. Nano Lett.

[19] Wang J., Scampicchio M., Laocharoensuk R., Valentini F., Gonzalez-Garcia O., Burdick J. (2006). Magnetic tuning of the electrochemical reactivity through controlled surface orientation of catalytic nanowires. J. Am. Chem. Soc.

[20] Wang J., Musameh M., Laocharoensuk R. (2005). Magnetic catalytic nickel particles for on-demand control of electrocatalytic processes. Electrochem. Comm.

[21] Katz E., Willner I. (2004). Magneto-stimulated hydrodynamic control of electrocatalytic and bioelectrocatalytic processes. J. Am. Chem. Soc.

[22] Yin Y., Alivisatos A.P. (2005). Colloidal nanocrystal synthesis and the organic−inorganic interface. Nature.

[23] Park H.Y., Schadt M.J., Wang L., Lim I.I.S., Njoaki P.N., Kim S.H., Jang M.J., Luo J., Zhong C.J. (2007). Fabrication of magnetic core@shell Fe oxide@Au nanoparticles for interfacial bioactivity and bio-separation. Langmuir.

[24] Toderas F., Baia M., Maniu D., Astilean S. (2008). Tuning the plasmon resonances of gold nanoparticles by controlling their size and shape. J. Optoelectron. Adv. M.

[25] Wei Y., Klajn R., Pinchuk A.O., Grzybowski B.A. (2008). Synthesis, shape control, and optical properties of hybrid Au/Fe_3_O_4_ “nanoflowers”. Small.

[26] Hickey S.G., Waurisch C., Rellinghaus B., Eychmuller A. (2008). Size and shape control of colloidally synthesized IV–VI nanoparticulate tin(II) sulfide. J. Am. Chem. Soc.

[27] Piccolo L., Valcarcel A., Bausach M., Thomazeau C., Uzio D., Berhault G. (2008). Tuning the shape of nanoparticles to control their catalytic properties: selective hydrogenation of 1,3-butadiene on Pd/Al_2_O_3_. Phys. Chem. Chem. Phys.

[28] Zhang Z., Wong L.M., Ong H.G., Wang X.J., Wang J.L., Wang S.J., Chen H., Wu T. (2008). Self-assembled shape - and orientation- controlled synthesis of nanoscale Cu_3_Si triangles, squares, and wires. Nano Lett.

[29] Grzelczak M., Perez-Juste J., Mulvaney P., Liz-Marzan L.M. (2008). Shape control in gold nanoparticle synthesis. Chem. Soc. Rev.

[30] Hachisu T., Yotsumoto T., Sugiyama A., Iida H., Nakanishi T., Asahi T., Osaka T. (2008). Effect of growth temperature on the shape and crystallinity of chemically produced Fe-Pt nanoparticles. Chem. Lett.

[31] Vassilieff T., Sutton A., Kakkar A.K. (2008). Shape control in silver metal nanoparticle construction using dumb-bell dendrimers. J. Mater. Chem.

[32] Kim M.H., Lim B., Lee E.P., Xia Y. (2008). Polyol synthesis of Cu_2_O nanoparticles: use of chloride to promote the formation of a cubic morphology. J. Mater. Chem.

[33] Somorjai G.A., Park J.Y. (2008). Colloid science of metal nanoparticle catalysts in 2D and 3D structures. challenges of nucleation, growth, composition, particle shape, size control and their influence on activity and selectivity. Top. Catal.

[34] Hofmann C., Rusakova I., Ould-Ely T., Prieto-Centurion D., Hartman K.B., Kelly A.T., Luttge A., Whitmire K.H. (2008). Shape control of new Fe_x_O-Fe_3_O_4_ and Fe1-yMnyO-Fe_3_-zMn_z_O_4_ nanostructures. Adv. Funct. Mater.

[35] Susut C., Nguyen T.D., Chapman G.B., Tong Y. (2008). Shape and size stability of Pt nanoparticles for MeOH electro-oxidation. Electrochim. Acta.

[36] Chou N.H., Ke X., Schiffer P., Schaak R.E. (2008). Room-temperature chemical synthesis of shape-controlled indium nanoparticles. J. Am. Chem. Soc.

[37] Kilin D.S., Prezhdo O.V., Xia Y. (2008). Shape - controlled synthesis of silver nanoparticles: Ab initio study of preferential surface coordination with citric acid. Chem. Phys. Lett.

[38] Scholes G.D. (2008). Controlling the optical properties of inorganic nanoparticles. Adv. Funct. Mater.

[39] Majewski P., Thierry B. (2008). Superparamagnetic magnetite (Fe_3_O_4_) nanoparticles for bio-applications. Recent Pat. Mater. Sci.

[40] Burda C., Chen X.B., Narayanan R., El-Sayed M.A. (2005). Chemistry and properties of nanocrystals of different shapes. Chem. Rev.

[41] Taborda A., Carvalho A. (2008). Superparamagnetic iron oxide nanoparticles - proton nuclear magnetic resonance dispersion curves. Eur. Phys. J. Appl. Phys.

[42] Park J.H., von Maltzahn G., Zhang L., Schwartz M.P., Ruoslahti E., Bhatia S.N., Sailor Michael J. (2008). Magnetic iron oxide nanoworms for tumor targeting and imaging. Adv. Mater.

[43] Zhai Y., Wang X., Wang X., Xie H., Gu H. (2008). Acute toxicity and irritation of water-based dextran – coated magnetic fluid injected in mice. J. Biomed. Mater. Res. A.

[44] Wu C.C., Lin L.Y., Lin L.C., Huang H.C., Yang Y.F., Liu Y.B., Tsai M.C., Gao Y.L., Wang W.C., Hung S.W., Yang S.Y., Horng H.E., Yang H.C., Tseng W.Y.I., Yeh H.I., Hsuan C.F., Lee T.L., Tseng W.K. (2008). Biofunctionalized magnetic nanoparticles for *in vitro* labeling and *in vivo* locating specific biomolecules. Appl. Phys. Lett.

[45] Yu J.H., Lee C.W., Im S.S., Lee J.S. (2003). Structure and magnetic properties of SiO_2_ coated Fe_2_O_3_ nanoparticles synthesized by chemical vapor condensation process. Rev. Adv. Mater. Sci.

[46] Sharma R., Malik R., Lamba S., Annapoorni S. (2008). Metal oxide/polyaniline nanocomposites: cluster size and composition dependent structural and magnetic properties. Bull. Mater. Sci.

[47] Wang Z., Bi H., Liu J., Sun T., Wu X. (2008). Magnetic and microwave absorbing properties of polyaniline / -Fe_2_O_3_ nanocomposite. J. Magn. Magn. Mater.

[48] Reddy K.R., Lee K.P., Iyengar A.G. (2007). Synthesis and characterization of novel conducting composites of Fe_3_O_4_ nanoparticles and sulfonated polyanilines. J. Appl. Polym. Sci.

[49] Jacobo S.E., Aphesteguy J.C., Lopez A.R., Schegoleva N.N., Kurlyandskaya G.V. (2007). Influence of the preparation procedure on the properties of polyaniline based magnetic composites. Eur. Polym. J.

[50] Zhao D.L., Zeng X.W., Xia Q.S., Tang J.T. (2007). Fe_3_O_4_/polyaniline nanoparticles with core-shell structure and their inductive heating property in AC magnetic field. Key Eng. Mater.

[51] Zhao D.L., Zhang H.L., Zeng X.W., Xia Q.S., Tang J.T. (2006). Inductive heat property of Fe_3_O_4_/polymer composite nanoparticles in an ac magnetic field for localized hyperthermia. Biomed. Mater.

[52] Dallas P., Moutis N., Devlin E., Niarchos D., Petridis D. (2006). Characterization, electrical and magnetic properties of polyaniline /maghemite nanocomposites. Nanotechnology.

[53] Long Y., Chen Z., Duvail J.L., Zhang Z., Wan M. (2005). Electrical and magnetic properties of polyaniline /Fe_3_O_4_ nanostructures. Phys. B.

[54] Lu X., Yu Y., Chen L., Mao H., Gao H., Wang J., Zhang W., Wei Y. (2005). Aniline dimer-COOH assisted preparation of well - dispersed polyaniline - Fe_3_O_4_ nanoparticles. Nanotechnology.

[55] Sharma R., Lamba S., Annapoorni S., Sharma P., Inoue A. (2005). Composition dependent magnetic properties of iron oxide - polyaniline nanoclusters. J. Appl. Phys.

[56] Lin H.A., Liu C.H., Huang W.C., Liou S.C., Chu M.W., Chen C.H., Lee J.F., Yang C.M. (2008). Novel magnetically separable mesoporous Fe_2_O_3_@SBA-15 nanocomposite with fully open mesochannels for protein immobilization. Chem. Mater.

[57] Souza D.M., Andrade A.L., Fabris J.D., Valerio P., Goes A.M., Leite M.F., Domingues R.Z. (2008). Synthesis and *in vitro* evaluation of toxicity of silica -coated magnetite nanoparticles. J. Non-Cryst. Solids.

[58] Zhang F., Wang C.C. (2008). Fabrication of one-dimensional iron oxide / silica nanostructures with high magnetic sensitivity by dipole-directed self-assembly. J. Phys. Chem. C.

[59] Lien Y.H., Wu T.M. (2008). Preparation and characterization of thermosensitive polymers grafted onto silica -coated iron oxide nanoparticles. J. Colloid Interface Sci.

[60] Gu R., Gong X., Jiang W., Hao L., Xuan S., Zhang Z. (2008). Synthesis and rheological investigation of a magnetic fluid using olivary silica -coated iron particles as a precursor. J. Magn. Magn. Mater.

[61] Hu S.H., Chen S.Y., Liu D.M., Hsiao C.S. (2008). Core/single-crystal-shell nanospheres for controlled drug release via a magnetically triggered rupturing mechanism. Adv. Mater.

[62] Li L., Choo E.S.G., Liu Z., Ding J., Xue J. (2008). Double-layer silica core-shell nanospheres with superparamagnetic and fluorescent functionalities. Chem. Phys. Lett.

[63] Heitsch A.T., Smith D.K., Patel R.N., Ress D., Korgel B.A. (2008). Multifunctional particles: Magnetic nanocrystals and gold nanorods coated with fluorescent dye-doped silica shells. J. Solid State Chem.

[64] Xu Q., Bian X.J., Li L.L., Hu X.Y., Sun M., Chen D., Wang Y. (2008). Myoglobin immobilized on Fe_3_O_4_@SiO_2_ magnetic nanoparticles: Direct electron transfer, enhanced thermostability and electroactivity. Electrochem. Comm.

[65] Shukoor M.I., Natalio F., Therese H.A., Tahir M.N., Ksenofontov V., Panthoefer M., Eberhardt M., Theato P., Schroeder H.C., Mueller W.E.G., Tremel W. (2008). Fabrication of a silica coating on magnetic γ-Fe_2_O_3_ nanoparticles by an immobilized enzyme. Chem. Mater.

[66] Wang S., Cao H., Gu F., Li C., Huang G. (2008). Synthesis and magnetic properties of iron / silica core/shell nanostructures. J. Alloy. Compd.

[67] Zhang M., Cushing B.L., O’Connor C.J. (2008). Synthesis and characterization of monodisperse ultra-thin silica-coated magnetic nanoparticles. Nanotechnology.

[68] Lei Z., Li Y., Wei X. (2008). A facile two-step modifying process for preparation of poly(SStNa)-grafted Fe_3_O_4_/SiO_2_ particles. J. Solid State Chem.

[69] Song Q., Fei H., Gang C., Shaoning Y., Jilie K. (2007). Magnetic assembled electrochemical platform using Fe_2_O_3_ filled carbon nanotubes and enzyme. Electrochem. Comm.

[70] Gorin D.A., Portnov S.A., Inozemtseva O.A., Luklinska Z., Yashchenok A.M., Pavlov A.M., Skirtach A.G., Moehwald H., Sukhorukov G.B. (2008). Magnetic / gold nanoparticle functionalized biocompatible microcapsules with sensitivity to laser irradiation. Phys. Chem. Chem. Phys.

[71] Wang L., Park H.Y., Lim S.I.I., Schadt M.J., Mott D., Luo J., Wang X., Zhong C.J. (2008). Core@shell nanomaterials: gold-coated magnetic oxide nanoparticles. J. Mater. Chem.

[72] Gole A., Stone J.W., Gemmill W.R., zur Loye H.C., Murphy C.J. (2008). Iron oxide coated gold nanorods: synthesis, characterization, and magnetic manipulation. Langmuir.

[73] Hien P., Thao T., Cao C., Sim S.J. (2008). Application of citrate-stabilized gold-coated ferric oxide composite nanoparticles for biological separations. J. Magn. Magn. Mater.

[74] Tang D., Yuan R., Chai Y. (2008). Magneto-controlled bioelectronics for the antigen-antibody interaction based on magnetic-core/ gold-shell nanoparticles functionalized biomimetic interface. Bioproc. Biosyst. Eng.

[75] Bao J., Chen W., Liu T., Zhu Y., Jin P., Wang L., Liu J., Wei Y., Li Y. (2007). Bifunctional Au-Fe_3_O_4_ Nanoparticles for Protein Separation. ACS Nano.

[76] Kouassi G.K., Wang P., Sreevatan S., Irudayaraj J. (2007). Aptamer-mediated magnetic and gold-coated magnetic nanoparticles as detection assay for prion protein assessment. Biotechnol. Progr.

[77] Xu Z., Hou Y., Sun S. (2007). Magnetic core/shell Fe_3_O_4_/Au and Fe_3_O_4_/Au/Ag nanoparticles with tunable plasmonic properties. J. Am. Chem. Soc.

[78] Lo C.K., Xiao D., Choi M.M.F. (2007). Homocysteine-protected gold-coated magnetic nanoparticles: synthesis and characterization. J. Mater. Chem.

[79] Lu Q.H., Yao K.L., Xi D., Liu Z.L., Luo X.P., Ning Q. (2006). A magnetic separation study on synthesis of magnetic Fe oxide core/Au shell nanoparticles. Nanoscience.

[80] Kim S.H., Kim M.J., Choa Y.H. (2007). Fabrication and estimation of Au-coated Fe_3_O_4_ nanocomposite powders for the separation and purification of biomolecules. Mater. Sci. Eng. A.

[81] Sun Q., Reddy B.V., Marquez M., Jena P., Gonzalez C., Wang Q. (2007). Theoretical study on gold-coated iron oxide nanostructure: Magnetism and bioselectivity for amino acids. J. Phys. Chem. C.

[82] Seino S., Kusunose T., Sekino T., Kinoshita T., Nakagawa T., Kakimi Y., Kawabe Y., Iida J., Yamamoto T.A., Mizukoshi Y. (2006). Synthesis of gold / magnetic iron oxide composite nanoparticles for biomedical applications with good dispersibility. J. Appl. Phys.

[83] Yu H., Chen M., Rice P.M., Wang S.X., White R.L., Sun S. (2005). Dumbbell-like bifunctional Au-Fe_3_O_4_ nanoparticles. Nano Lett.

[84] Chang Q., Zhu L., Yu C., Tang H. (2008). Synthesis and properties of magnetic and luminescent Fe_3_O_4_/SiO_2_/Dye/SiO_2_ nanoparticles. J. Lumin.

[85] Qiu J., Peng H., Liang R. (2007). Ferrocene modified Fe_3_O_4_@SiO_2_ magnetic nanoparticles as building blocks for construction of reagentless enzyme-based biosensors. Electrochem. Comm.

[86] Yoon T.J., Kim J.S., Kim B.G., Yu K.N., Cho M.H., Lee J.K. (2005). Multifunctional nanoparticles possessing a “Magnetic motor effect” for drug or gene delivery. Angew. Chem. Int. Ed.

[87] Qiu G.M., Xu Y.Y., Zhu B.K., Qiu G.L. (2005). Novel, fluorescent, magnetic, polysaccharide-based microsphere for orientation, tracing, and anticoagulation: preparation and characterization. Biomacromolecules.

[88] Teng X., Yang H. (2004). Effects of surfactants and synthetic conditions on the sizes and self-assembly of monodisperse iron oxide nanoparticles. J. Mater. Chem.

[89] Gaponik N., Radtchenko I.L., Sukhorukov G.B., Rogach A.L. (2004). Luminescent polymer microcapsules addressable by a magnetic field. Langmuir.

[90] Zhu Y., Hong D., Yang X., Hu Y. (2003). Preparation and characterization of core-shell monodispersed agnetic silica microspheres. Colloids Surf. A.

[91] Claesson E.M., Philipse A.P. (2005). Monodisperse magnetizable composite silica spheres with tunable dipolar interactions. Langmuir.

[92] Yang C., Guan Y., Xing J., Liu J., Shan G., An Z., Liu H. (2005). Preparation of magnetic polystyrene microspheres with a narrow size distribution. AIChE J.

[93] Isher B.R., Eisler H.J., Stott N.E., Bawendi M.G. (2004). Emission intensity dependence and single-exponential behavior in single colloidal quantum dot fluorescence lifetimes. J. Phys. Chem. B.

[94] ankhurst Q.A., Connolly J., Jones S.K., Dobson J. (2003). Applications of magnetic nanoparticles in biomedicine. J. Phys. D: Appl. Phys.

[95] nker J.N., Kopelman R. (2003). Magnetically modulated optical nanoprobes. Appl. Phys. Lett.

[96] ang H.H., Zhang S.Q., Chen X.L., Zhuang Z. X, Xu J.G., Wang X.R. (2004). Magnetite-containing spherical silica nanoparticles for biocatalysis and bioseparations. Anal. Chem.

[97] Lee J., Lee Y., Youn J.K., Na H.B., Yu T., Kim H., Lee S.M., Koo Y.M., Kwak J.H., Park H.G., Chang H.N., Hwang M., Park J.G., Kim J., Hyeon T. (2008). Simple synthesis of functionalized superparamagnetic magnetite/silica core/shell nanoparticles and their application as magnetically separable high-performance biocatalysts. Small.

[98] Lee D.C., Mikulec F.V., Pelaez J.M., Koo B., Korgel B.A. (2006). Synthesis and magnetic properties of silica-coated FePt nanocrystals. J. Phys. Chem. B.

[99] Lee I.S., Lee N., Park J., Kim B.H., Yi Y.W., Kim T., Kim T.K., Lee I.H., Paik S.R., Hyeon T. (2006). Ni/NiO core/shell nanoparticles for selective binding and magnetic separation of histidine-tagged proteins. J. Am. Chem. Soc ..

[100] Yi D.K., Lee S.S., Papaefthymiou G.C., Ying J.Y. (2006). Nanoparticle architectures templated by SiO_2_/Fe_2_O_3_ nanocomposites. Chem. Mater.

[101] Yi D.K., Selvan S.T., Lee S.S., Papaefthymiou G.C., Kundaliya D., Ying J.Y. (2005). Silica-coated nanocomposites of magnetic nanoparticles and quantum dots. J. Am. Chem. Soc.

[102] Zhao W., Gu J., Zhang L., Chen H., Shi J. (2005). Fabrication of uniform magnetic nanocomposite spheres with a magnetic core/mesoporous silica shell structure. J. Am. Chem. Soc.

[103] Trindade T., O'Brien P., Pickett N.L. (2001). Nanocrystalline semiconductors: Synthesis, properties, and perspectives. Chem. Mater.

[104] Lue J.T. (2001). A review of characterization and physical property studies of metallic nanoparticles. J. Phys. Chem. Solids.

[105] Grieve K., Mulvaney P., Grieser F. (2000). Synthesis and electronic properties of semiconductor nanoparticles/quantum dots. Curr. Opin. Colloid Interface Sci.

[106] Schwerdtfeger P. (2003). Gold goes nano- from small clusters to low-dimensional assemblies. Angew. Chem. Int. Ed.

[107] Brust M., Kiely C.J. (2002). Some recent advances in nanostructure preparation from gold and silver particles: a short topical review. Colloids Surf. A.

[108] McConnell W.P., Novak J.P., Brousseau L.C., Fuierer R.R., Tenent R.C., Feldheim D.L. (2000). Electronic and optical properties of chemically modified metal nanoparticles and molecularly bridged nanoparticle arrays. J. Phys. Chem. B.

[109] Gangopadhyay R., De A. (2000). Conducting polymer nanocomposites: A brief overview. Chem. Mater.

[110] Katz E., Shipway A.N., Willner I., Schmid G. (2003). Nanoparticles-From Theory to Applications.

[111] Niemeyer C.M. (2001). Nanoparticles, proteins, and nucleic acids: Biotechnology meets materials science. Angew. Chem. Int. Ed.

[112] Niemeyer C.M. (2003). Functional hybrid devices of proteins and inorganic nanoparticles. Angew. Chem. Int. Ed.

[113] Parak W.J., Gerion D., Pellegrino T., Zanchet D., Micheel C., Williams S.C., Boudreau R., Le Gros M.A., Larabell C.A., Alivisatos A.P. (2003). Biological applications of colloidal nanocrystals. Nanotechnology.

[114] Csaki A., Maubach G., Born D., Reichert J., Fritzsche W. (2002). DNA-based molecular nanotechnology. Single Molecules.

[115] Hirsch R., Katz E., Willner I. (2000). Magneto-Switchable Bioelectrocatalysis. J. Am. Chem. Soc.

[116] Urban M., Moller R., Fritzsche W. (2003). A paralleled readout system for an electrical DNA-hybridization assay based on a microstructured electrode array. Rev. Sci. Instrum.

[117] Katz E., Willner I. (2004). Nanobiotechnology: integrated nanoparticle-biomolecule hybrid systems: Synthesis, properties, and applications. Angew. Chem. Int. Ed.

[118] Bartlett P.N., Tebbutt P., Whitaker R.G. (1991). Kinetic aspects of the use of modified electrodes and mediators in bioelectrochemistry. Prog. React. Kinet.

[119] Bardea A., Katz E., Bueckmann A.F., Willner I. (1997). NAD^+^-dependent enzyme electrodes: Electrical contact of cofactor-dependent enzymes and electrodes. J. Am. Chem. Soc.

[120] Zayats M., Katz E., Willner I. (2002). Electrical contacting of flavoenzymes and NAD(P)^+^-dependent enzymes by reconstitution and affinity interactions on phenylboronic acid monolayers associated with Au-electrodes. J. Am. Chem. Soc.

[121] Buckmann A.F., Wray V., Stocker A. (1997). Synthesis of N6-(2-aminoethyl)-FAD, N6-(6-carboxyhexyl)-FAD, and related compounds. Methods Enzymol.

[122] Katz E., Sheeney-Haj-Ichia L., Buckmann A.F., Willner I. (2002). Dual biosensing by magneto-controlled bioelectrocatalysis. Angew. Chem. Int. Ed.

[123] Georgelin T., Moreau B., Bar N., Villemin D., Cabuil V., Horner O. (2008). Functionalization of Fe_2_O_3_ nanoparticles through the grafting of an organophosphorous ligand. Sens. Actuat. B.

[124] Yong Y., Bai Y., Li Y., Lin L., Cui Y., Xia C. (2008). Preparation and application of polymer-grafted magnetic nanoparticles for lipase immobilization. J. Magn. Magn. Mater.

[125] Li G.Y., Huang K.L., Jiang Y.R., Yang D.L., Ding P. (2008). Preparation and characterization of Saccharomyces cerevisiae alcohol dehydrogenase immobilized on magnetic nanoparticles. Int. J. Biol. Macromol.

[126] Yu C.C., Lin P.C., Lin C.C. (2008). Site-specific immobilization of CMP-sialic acid synthetase on magnetic nanoparticles and its use in the synthesis of CMP-sialic acid. Chem. Commun.

[127] Fu X.H. (2008). Magnetic-controlled non-competitive enzyme-linked voltammetric immunoassay for carcinoembryonic antigen. Biochem. Eng. J.

[128] Hung C.W., Holoman T.R.P., Kofinas P., Bentley W.E. (2008). Towards oriented assembly of proteins onto magnetic nanoparticles. Biochem. Eng. J.

[129] Liu J., Lin S., Qi D., Deng C., Yang P., Zhang X. (2007). On-chip enzymatic microreactor using trypsin-immobilized superparamagnetic nanoparticles for highly efficient proteolysis. J. Chromatogr. A.

[130] Shukoor M.I., Natalio F., Tahir M.N., Ksenofontov V., Therese H.A., Theato P., Schroeder H.C., Mueller W.E.G., Tremel W. (2007). Superparamagnetic-Fe_2_O_3_ nanoparticles with tailored functionality for protein separation. Chem. Commun.

[131] Li Y., Yan B., Deng C., Yu W., Xu X., Yang P., Zhang X. (2007). Efficient on-chip proteolysis system based on functionalized magnetic silica microspheres. Proteomics.

[132] Liang Y.Y., Zhang L.M., Li W., Chen R.F. (2007). Polysaccharide-modified iron oxide nanoparticles as an effective magnetic affinity adsorbent for bovine serum albumin. Colloid Polym. Sci.

[133] Kim M.J., An G.H., Choa Y.H. (2007). Functionalization of magnetite nanoparticles for protein immobilization. Diffus. Defect Data, Pt. B.

[134] Liang Y.Y., Zhang L.M. (2007). Bioconjugation of papain on superparamagnetic nanoparticles decorated with carboxymethylated chitosan. Biomacromolecules.

[135] Lang C., Schueler D., Faivre D. (2007). Synthesis of magnetite nanoparticles for bio- and nanotechnology: genetic engineering and biomimetics of bacterial magnetosomes. Macromol. Biosci.

[136] Hong J., Gong P.J., Yu J.H., Xu D.M., Sun H.W., Yao S. (2006). Conjugation of chymotrypsin on a polymeric hydrophilic nanolayer covering magnetic nanoparticles. J. Mol. Catal. B: Enzym.

[137] Bilkova Z., Slovakova M., Minc N., Futterer C., Cecal R., Horak D., Benes M., le Potier I., Krenkova J., Przybylski M., Viovy J.L. (2006). Functionalized magnetic micro- and nanoparticles: optimization and application to chip tryptic digestion. Electrophoresis.

[138] Rossi L.M., Quach A.D., Rosenzweig Z. (2004). Glucose oxidase- magnetite nanoparticle bioconjugate for glucose sensing. Anal. Bioanal.Chem.

[139] Hong J., Gong P., Xu D., Dong L., Yao S. (2007). Stabilization of chymotrypsin by covalent immobilization on amine-functionalized superparamagnetic nanogel. J. Biotechnol..

[140] Hong J., Xu D., Gong P., Yu J., Ma H., Yao S. (2008). Covalent-bonded immobilization of enzyme on hydrophilic polymer covering magnetic nanogels. Microporous Mesoporous Mater.

[141] Wei C., Yang M., Hu J., Li Q. (2007). Electrocatalysis of horseradish peroxidase immobilized on cobalt nanoparticles modified ITO electrode. Anal. Lett.

[142] Sharma A., Qiang Y., Antony J., Meyer D., Kornacki P., Paszczynski A. (2007). Dramatic increase in stability and longevity of enzymes attached to monodispersive iron nanoparticles. IEEE Trans. Magn.

[143] Dyal A., Loos K., Noto M., Chang S.W., Spagnoli C., Shafi K.V.P.M., Ulman A., Cowman M., Gross R.A. (2003). Activity of candida rugosa lipase immobilized on gamma-Fe_2_O_3_ magnetic nanoparticles. J. Am. Chem. Soc.

[144] Wang T.H., Lee W.C. (2003). Immobilization of proteins on magnetic nanoparticles. Biotechnol. Bioprocess Eng.

[145] Kim J., Lee J., Na H.B., Kim B.C., Youn J.K., Kwak J.H., Moon K., Lee E., Kim J., Park J., Dohnalkova A., Park H.G., Gu M.B., Chang H.N., Grate J.W., Hyeon T. (2005). A magnetically separable, highly stable enzyme system based on nanocomposites of enzymes and magnetic nanoparticles shipped in hierarchically ordered, mesocellular, mesoporous silica. Small.

[146] Li Y., Xu X., Deng C., Yang P., Zhang X. (2007). Immobilization of trypsin on superparamagnetic nanoparticles for rapid and effective proteolysis. J. Proteome. Res.

[147] Jeng J., Lin M.F., Cheng F.Y., Yeh C.S., Shiea J. (2007). Using high-concentration trypsin-immobilized magnetic nanoparticles for rapid in situ protein digestion at elevated temperature. Rapid Commun. Mass Spectrom.

[148] Hong J., Xu D., Gong P., Sun H., Dong L., Yao S. (2007). Covalent binding of chymotrypsin on the magnetic nanogels covered by amino groups. J. Mol. Catal. B: Enzym ..

[149] Zavisova V., Koneracka M., Tomasovicova N., Kopcansky P., Timko M. (2006). Some immobilization modes of biologically active substances to fine magnetic particles. Z. Phys. Chem.

[150] Liu Z., Wang J., Xie D., Chen G. (2008). Polyaniline-coated Fe_3_O_4_ nanoparticle-carbon-nanotube composite and its application in electrochemical biosensing. Small.

[151] Kuroiwa T., Noguchi Y., Nakajima M., Sato S., Mukataka S., Ichikawa S. (2008). Production of chitosan oligosaccharides using chitosanase immobilized on amylose-coated magnetic nanoparticles. Process Biochem.

[152] Mahmood I., Guo C., Xia H., Ma J., Jiang Y., Liu H. (2008). Lipase Immobilization on oleic acid-pluronic (L-64) block copolymer coated magnetic nanoparticles, for hydrolysis at the oil/water interface. Ind. Eng. Chem. Res.

[153] Shamim N., Hong L., Hidajat K., Uddin M.S. (2007). Thermosensitive polymer coated nanomagnetic particles for separation of bio-molecules. Sep. Purif. Technol.

[154] Peng Z.G., Hidajat K., Uddin M.S. (2005). Selective and sequential adsorption of bovine serum albumin and lysozyme from a binary mixture on nanosized magnetic particles. J. Colloid Interface Sci.

[155] Kaushik A., Khan R., Solanki P.R., Pandey P., Alam J., Ahmad S., Malhotra B.D. (2008). Iron oxide nanoparticles–chitosan composite based glucose biosensor. Biosens. Bioelectron.

[156] Johnson A.K., Zawadzka A.M., Deobald L.A., Crawford R.L., Paszczynski A.J. (2008). Novel method for immobilization of enzymes to magnetic nanoparticles. J. Nanopart. Res.

[157] Naik R.R., Brott L.L., Clarson S.J., Stone M.O. (2002). Silica-precipitating peptides isolated from a combinatorial phage display peptide library. J. NanoSci. Nanotechno.

[158] Brown S. (1992). Engineered iron oxide-adhesion mutants of the *Escherichia coli* phage lambda receptor. Proc. Nat. Acad. Sci. USA.

[159] Istamboulie G., Andreescu S., Marty J.L., Noguer T. (2007). Highly sensitive detection of organophosphorus insecticides using magnetic microbeads and genetically engineered acetylcholinesterase. Biosens. Bioelectron.

[160] Elyacoubia A., Zayeda S.I.M., Blankert B., Kauffmann J.M. (2006). Development of an amperometric enzymatic biosensor based on gold modified magnetic nanoporous microparticles. Electroanalysis.

[161] Nomura A., Mehdi S.S.O., Kauffmann J.M. (2004). Preparation, Characterization, and application of an enzyme-immobilized magnetic microreactor for flow injection analysis. Anal. Chem.

[162] Liu Z., Liu Y., Yang H., Yang Y., Shen G., Yu R. (2005). A phenol biosensor based on immobilizing tyrosinase to modified core-shell magnetic nanoparticles supported at a carbon paste electrode. Anal. Chim. Acta.

[163] Rossi L.M., Quach A.D., Rosenzweig Z. (2004). Glucose oxidase–magnetite nanoparticle bioconjugate for glucose sensing. Anal. Bioanal.Chem.

[164] Ivanova V., Hristov J., Dobreva E., Al-Hassan Z., Penchev I. (1996). Performance of a magnetically stabilized bed reactor with immobilized yeast cells. Appl. Biochem. Biotechnol.

[165] Hsing I.M., Xu Y., Zhao W.T. (2007). Micro- and nano- magnetic particles for applications in biosensing. Electroanalysis.

[166] Li J.P., Gao H.D. (2008). A renewable potentiometric immunosensor based on Fe_3_O_4_ nanoparticles immobilized anti-IgG. Electroanalysis.

[167] Li Z.M., Yang H.F., Li Y.F., Liu Y.L., Shen G.L., Yu R.Q. (2006). Core-shell magnetic nanoparticles applied for immobilization of antibody on carbon paste electrode and amperometric immunosesning. Sens. Actuat. B.

[168] Santandreu M., Sole S., Fabregas E., Alegret S. (1998). Development of electrochemical immunosensing systems with renewable surfaces. Biosens. Bioelectron..

[169] Sole S., Alegret S., Cespedes F., Fabregas E. (1998). Flow injection immunoanalysis based on a magnetoimmunosensor system. Anal. Chem.

[170] Gehring A.G., Brewster J.D., Irwin P.L., Tu S.I., Van Houten L.J. (1999). 1-Naphtyl phosphate as an enzymatic substrate for enzyme-linked immunomagnetic electrochemistry. J. Electroanal. Chem.

[171] Perez F.G., Mascini M., Tothil I.E., Turner A.P.F. (1998). Immunomagnetic separation with mediated flow injection analysis amperometric detection of viable *Escherichia coli* O157. Anal. Chem.

[172] Dequaire M., Degrand C., Limoges B. (1999). An immunomagnetic electrochemical sensor based on a perfluorosulfonate-coated screen-printed electrode for the determination of 2,4-dichlorooxyacetic acid. Anal. Chem.

[173] Zacco E., Pividori M.I., Alegret S. (2006). Electrochemical magnetoimmunosensing strategy for the detection of pesticides residues. Anal. Chem.

[174] Ghering A.G., Crawford C.G., Mazenko R.S., VanHouten L.J., Brewster J.D. (1996). Enzyme-linked immunomagnetic electrochemical detection of Salmonella typhimurium. J. Immunol. Methods.

[175] Helali S., Martelet C., Abdelghani A., Maaref M.A., Jafrezic-Renault N. (2006). A disposable immunomagnetic electrochemical sensor based on functionalised magnetic beads on gold surface for the detection of atrazine. Electrochim. Acta.

[176] Varshney M., Li Y.B. (2007). Interdigitated array microelectrode based impedance biosensor coupled with magnetic nanoparticle – antibody conjugates for detection of *Escherichia coli* O157:H7 in food samples. Biosens. Bioelectron.

[177] Su X.L., Li Y.B. (2005). A QCM immunosensor for Salmonella detection with simultaneous measurements of resonant frequency and motional resistance. Biosens. Bioelectron.

[178] Centi S., Laschi S., Franek M., Mascini M. (2005). A disposable immunomagnetic electrochemical sensor based on functionalised magnetic beads and carbon-based screen-printed electrodes (SPCEs) for the detection of polychlorinated biphenyls (PCBs). Anal. Chim. Acta.

[179] Ho K.C., Tsai P.J., Lin Y.S., Chen Y.C. (2004). Using biofunctionalized nanoparticles to probe pathogenic bacteria. Anal. Chem.

[180] Gu H., Xu K., Xu C., Xu B. (2006). Biofunctional magnetic nanoparticles for protein separation and pathogen detection. Chem. Comm.

[181] Palacek E., Fojta M., Jelen F. (2002). New approaches in the developement of DNA sensors: hybridization and electrochemical detection of DNA and RNA at two different surfaces. Bioelectrochemistry.

[182] Wang J., Kawde A.N., Erdem A., Salazar M. (2001). Magnetic bead-based label-free electrochemical detection of DNA hybridization. Analyst.

[183] Kerman K., Matsubara Y., Morita Y., Takamura Y. (2004). Peptide nucleic acid modified magnetic beads for intercalator based electrochemical detection of DNA hybridization. Sci. Technol. Adv. Mater.

[184] Liu R. H, Yang J.N., Lenigk R., Bonanno J., Grodzinski P. (2004). Self-contained, fully integrated biochip for sample preparation, polymerase chain reaction amplification, and DNA microarray detection. Anal. Chem.

[185] Palecek E., Fojita M. (2007). Magnetic beads as versatile tools for electrochemical DNA and protein biosensing. Talanta.

[186] Erdem A., Sayar F., Karadeniz H., Guven G, Oszos M., Piskin E. (2007). Development of streptavidin carrying magnetic nanoparticles and their applications in electrochemical nucleic acid sensor systems. Electroanalysis.

[187] Grancharov S.G., Zeng H., Sun S.H., Wang S.X., O’Brien S., Murray C.B., Kirtley J.R., Held G.A. (2005). Bio-functionalization of monodisperse magnetic nanoparticles and their use as biomolecular labels in a magnetic tunnel junction based sensor. J. Phys. Chem. B.

[188] Robinson D.B., Persson H.H.J., Zeng H., Li G.X., Pourmand N., Sun S.H., Wang S.X. (2005). DNA-functionalized MFe_2_O_4_ (M = Fe, Co, or Mn) nanoparticles and their hybridization to DNA-functionalized surfaces. Langmuir.

[189] Stoeva S.I., Huo F., Lee J.S., Mirkin C.A. (2005). Three-layer composite magnetic nanoparticle probes for DNA. J. Am. Chem. Soc.

[190] Lermo A., Campoy S., Barbe J., Hernandez S., Alegret S., Pividori M.I. (2007). In situ DNA amplification with magnetic primers for the electrochemical detection of food pathogens. Biosens. Bioelectron.

[191] Loaiza O.A., Campuzano S., Pedrero M., Pedro G., Pingarro′n J.M. (2009). Ultrasensitive detection of coliforms by means of direct asymmetric PCR combined with disposable magnetic amperometric genosensors. Analyst.

